# Bacteriological and Molecular Identification of *Bartonella* Species in Cats from Different Regions of China

**DOI:** 10.1371/journal.pntd.0001301

**Published:** 2011-09-06

**Authors:** Congli Yuan, Caixia Zhu, Yanbing Wu, Xueying Pan, Xiuguo Hua

**Affiliations:** 1 Shanghai Jiaotong University, Shanghai, People's Republic of China; 2 Shanghai Key Laboratory of Veterinary Medicine, Shanghai, People's Republic of China; 3 Inner Mongolia Agricultural University, Hohhot, Inner Mongolia, People's Republic of China; University of Texas Medical Branch, United States of America

## Abstract

With the improvements in diagnostic techniques, *Bartonella henselae* (*B*. *henselae*) infection has recently been recognized to cause a widening spectrum of diseases. Cats are the natural reservoir hosts of *B*. *henselae*. The current study aims to investigate the prevalence of *B*. *henselae* infection in the cat populations in China. Polymerase chain reaction (PCR) and bacterial cultures confirm that 12.7% of the tested cats were positive for the infection. Old age and outdoor exposure were statistically associated with the infection. Multilocus sequence typing and eBURST analysis of the cat isolates collected in the present study show that 65.4% of the isolates belong to sequence type 1 (ST1). Three new STs (ST16–18) were identified in Midwestern China. These results may aid our understanding of the population structure of *B*. *henselae* in China and the relationship between human and cat strains in subsequent studies.

## Author Summary

*Bartonella* infection (*Bartonella henselae* in paticular) is responsible for a widening spectrum of human diseases. Cats are thought as the major source of human infections. In most of the developing countries, however, this infection lacks recognition especially in clinicians. Some well known bartonelloses, like cat-scratch disease, bacillary angiomatosis, and infective endocarditis had not been clinically noticed in these countries. In the present study, the prevalence of *Bartonella* infection in cat populations was investigated in mainland China. As a result, 12.7% of cats were confirmed positive with *Bartonella* infection. Factors of age, gender, outdoor experience, and geographic location were analyzed in association with infection. The authors found old cats and stray cats are susceptible populations. Some new molecular types are prevalent in Mid-Western China. Inclusion of these types also changed the characteristics of original molecular structure. This research provides the first molecular investigation of *Bartonella* infection in cats in China, and promotes our understanding of the relationship between human and cat strains in subsequent studies.

## Introduction

*Bartonella* is a small, fastidious, intracellular Gram-negative bacterium that has been recently identified in a wide range of domestic and wild mammals. The genus *Bartonella* has expanded from *Bartonella bacilliformis* to at least 20 recognized species during the past century, of which at least 10 species and subspecies are known or suspected to be pathogenic to humans [1], [2]. Of these, *B*. *henselae* is the most common *Bartonella* infection worldwide, resulting in cat-scratch disease (CSD), bacillary angiomatosis (BA), endocarditis, and neuroretinitis [3], [4]. Cats are the major reservoir hosts of *B*. *henselae*. Two main *B*. *henselae* 16S rRNA genotypes have been identified and designated as Houston-1 and Marseille. Marseille is the dominant type in cats in the United States, Europe, and Australia, whereas Houston-1 is dominant in Asia [5], [6]. In China, only rodent-associated bartonellosis has been well documented [7], [8]. However, little is known about the prevalence of *B*. *henselae* infection in the cat populations in China. Thus, the current study aims to evaluate *B*. *henselae* infection in Chinese cats using bacterial cultures, PCR detection, and sequence typing.

## Materials and Methods

### Ethics statement

This study was performed in accordance with the recommendations in the Guide for the Care and Use of Laboratory Animals of the Ministry of Health, China. The current study was approved by the Shanghai Animal Management Committee. (Permit Number:SYXK2007-0025).

### Sampling

**Figure 1:**
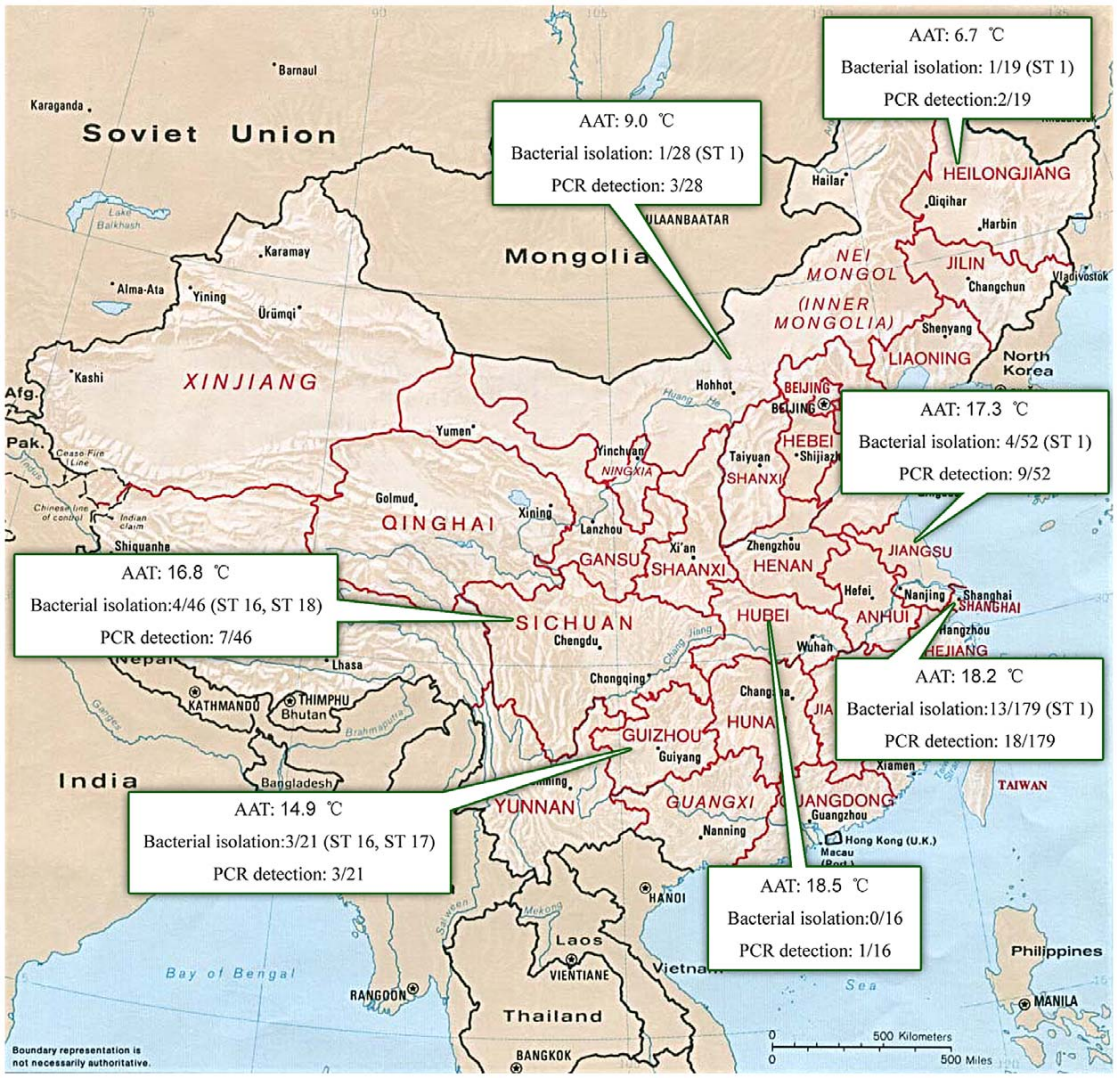
Geographic location of tested cats. The results of infection prevalence and the STs of isolates are specified. AAT means the average annual temperature.

Three hundred fifteen blood samples from pet cats and 46 samples from stray cats were collected from March to September in 2010. The geographic locations are specified in Figure 1. The mean age of the pet cats was 3.1 years (in the range of 4 months to 9 years). The age of the stray cats was unavailable. Of the 361 cats, 209 were male and 152 were female. About 2 ml of EDTA-anticoagulated blood was collected from each cat by venipuncture. One ml was stored at −80°C for subsequent culture. The remaining blood was centrifuged and the cell pellet was collected for PCR detection.

### Bacterial culture

The freeze-thawed blood samples (500 µl) were plated in Columbia agar containing 10% defibrinated sheep blood. The plates were incubated at 35°C in a 5% CO_2_, water-saturated atmosphere. Reference *B*. *henselae* strain (Houston-1) was cultured as the control. The plates were examined daily for 5 weeks for evidence of growth. Samples with bacterial contamination were immediately discarded. Positive cultures were further confirmed by PCR detection.

### Species-differentiated PCR detection

DNA from the cell pellet of the collected samples was extracted using the QIAamp DNA Blood Mini Kit according to the manufacturer's instructions. PCR primers (5′-CTCTTTCTTCAGATGATGATCC-3′ and 5′-AACCAACTGAGCTACAAGCCCT-3′) resulted in amplified products of approximately 202 (*B*. *bacilliformis*), 145 (*B*. *clarridgeiae*), 232 (*B*. *elizabethae*), 163 (*B*. *henselae*), 148 (*B*. *quintana*), and 251 bp (*B*. *vinsonii* subsp. *berkhoffii*), which are in accordance with previous study [9]. The DNA fragment was amplified with a 10 min extension step after 36 cycles of 94°C for 1 min, 60°C for 1 min, and 72°C for 1 min,. For better differentiation, 4.5% agarose gel electrophoresis was performed. DNA of *B*. *henselae* (Houston-1), *B*. *bacilliformis* (KC584), *B*. *quintana* (90–268) and *B*. *clarridgeiae* (NCSU 94-F40) was used as the control. Amplifications other than *B*. *henselae* from tested blood samples were further sequenced.

### Multilocus sequence typing (MLST) and data analysis

The *B*. *henselae* colonies isolated in the present study were scraped from the plates. DNA was extracted using QIAamp DNA Mini Kit. The PCR procedure and primers used are in accordance with previous studies [10], [11]. Nucleotide sequence data were collected from all *B*. *henselae* isolates of nine genetic loci (16SrRNA [*rrs*], *bat*R, *glt*A, *gro*EL, *fts*Z, *nlp*D, *rib*C, *rpo*B, and eno). The DNA amplifications were purified and sequenced for both strands. All DNA sequences were manually analyzed for polymorphism.

The nucleotide sequences were analyzed using the DNASTAR Lasergene software package 7 (DNASTAR, Madison, USA). New alleles were confirmed by re-sequencing. Alleles and sequence types (STs) were assigned in accordance with the published data [11]. New allelic combinations encountered for the first time in the current study were assigned to new STs in the order of detection. The definition of clonal complexes and the examination of relationships between STs within clonal complexes were carried out using BURST v3 analysis (http://burst.mlst.net).

### Statistical analysis

Statistical analysis of the association between *B*. *henselae* infection in cats and gender, age, and geographic region was performed using a χ^2^-test. A value of *P*<0.05 was considered significant.

### Nucleotide sequence accession numbers

New *rrs* and *bat*R alleles were obtained, and they were designated as *rrs* alleles 3 and 4, and *bat*R allele 5. The sequences of *rrs* alleles 3 and 4, as well as that of *bat*R allele 5 were deposited in GenBank under accession numbers JF819177 (*rrs* allele 3), JF819178 (*rrs* allele 4), and JF819179 (*bat*R allele 5).

## Results

**Figure 2:**
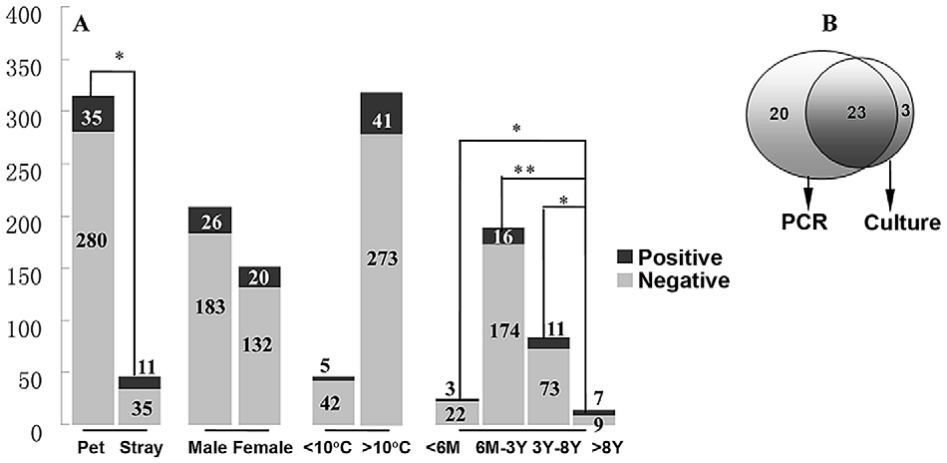
Infection prevalence and association with outdoor frequency, gender, age, and geographic origin. (A) The risk factors associated with *B*. *henselae* infection: “*” *P*<0.05; “**” *P*<0.01. (B) The results confirmed by PCR and bacterial isolation.

**Figure 3:**
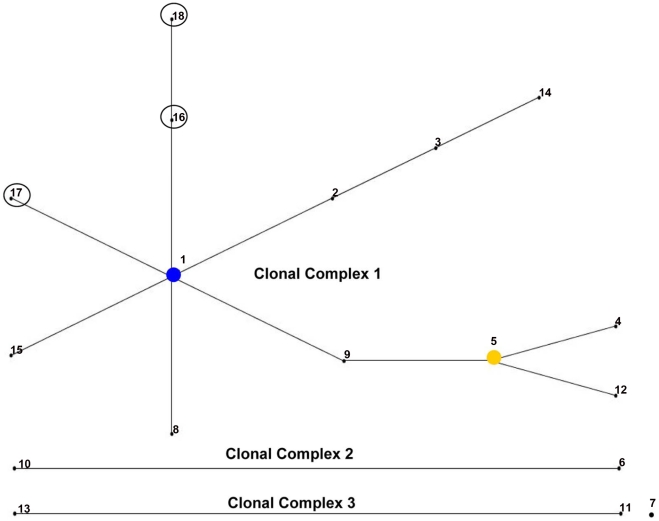
Phylogenetic relationship between the different *B*. *henselae* STs as determined by eBURST. The new STs identified in the present study are closely related to ST1. The inclusion of the new STs altered the structure of the phylogenetic tree: ST1 is defined as the primary founder, whereas ST5 is defined as a subgroup founder.

During the five-week bacterial isolation, two samples from pet cats and three samples from stray cats were discarded because of bacterial or fungi contamination. A total of 26 *B*. *henselae* strains were successfully isolated from 5.8% of the pet cats (18/313) and 18.6% of the stray cats (8/43), confirming that bacteremia more frequently occurs in stray cats (*P*<0.05). The average growth time for bacterial isolation was 7.6 days, with the shortest at 4 days and the longest at 23 days. The CFU/ml value of positive samples varied significantly from 4 to 2.4×10^2^. PCR detection using species-differentiated primers confirmed that 10.5% of the pet cats (33/315) and 21.7% of the stray cats (10/46) were PCR positive for *B*. *henselae*. Three positive samples with low bacterial loads that were confirmed by culture isolation (<6 CFU/ml) were PCR negative. *B*. *clarridgeiae* DNA was detected in four stray cats. Of these, two cats were co-infected with *B*. *henselae*. In total, 46 cats were confirmed infected with *B*. *henselae* (Figure 2). Statistical analysis shows no significant difference in the infection between male (12.4% positive) and female cats (13.1% positive). However, *B*. *henselae* infection was more prevalent in old animals (Figure 2). Based on the average annual temperature, the regions where the tested cats were located are defined as cold and tropical zones. However, climate difference was not statistically associated with infection, although a mild decrease in the prevalence of infection was observed in the colder regions in the current study (Figures 1 and 2). MLST analysis confirmed seventeen of the isolates in current study (65.4%) belong to the ST1 type. Two new *rrs* alleles (alleles 3 and 4) were obtained from seven and two isolates, respectively. The rrs allele 3 is caused by two nucleotide variations at positions 1414626 (T instead of C) and 1414146 (A instead T) of the *B*. *henselae* Houston-1 chromosome, whereas allele 4 consists of a single nucleotide insert based on the allele 3 sequence at position 1414562 of the *B*. *henselae* Houston-1 chromosome. A new allele of the *bat*R gene, with a single nucleotide variation at position 85173 (G instead of A) of the *B*. *henselae* Houston-1 chromosome, was designated as batR allele 5. Thus, three STs were encountered for the first time in the current study and designated as ST16 (seven isolates), ST 17 (two isolates), and ST18 (one isolate), in the order of detection (Table 1). The ST relationship was analyzed by eBURST. All newly identified STs were assigned to the previously described single clonal complex, Group 1 (Figure 3).

**Table 1:** Allele profile of new STs identifed in the present study.

ST	No of isolates	*rrs*	*batR*	*gltA*	*ftsZ*	*groEL*	*nlpD*	*ribC*	*rpoB*	Distribution
16	7	3	1	1	1	1	1	1	1	SiChuan,Guizhou
17	2	4	1	1	1	1	1	1	1	Guizhou
18	1	3	5	1	1	1	1	1	1	SiChuan

## Discussion

*B*. *henselae* has been found responsible for a widening range of clinical syndromes, particularly those of CSD, BA, endocarditis, and neuroretinitis [12]. Cats are the major reservoir host of *B*. *henselae*. In China, several studies confirmed a 9.6%–19.6% seropositivity to *B*. *henselae* infection in humans using immunofluorescence assays [13], [14]. However, little is known about the status of infections in cats in China. The homemade ELISA using whole bacterial antigens was not used in the present epidemiological study because of the inadequate number of control serum samples for accurate evaluation. Moreover, the high seroprevalence in cats renders the serologic testing impractical for infection diagnosis. Therefore, PCR combined with bacterial culture, was utilized in the present investigation.

Overall, 46 cats were confirmed PCR and/or culture positive. Of these, 26 bacteremic cats (7.3%) were determined by culture isolation. Previous studies have confirmed that *Bartonella* seroprevalences are higher in older cats than in younger animals, whereas the prevalence of bacteremia is higher in younger animals [15]. However, bacteremia was more frequently identified in cats older than 8 years in the present study. Since the sample size of old cats was relative small in the current study, the association of high prevalence with old age need further confirmation. The seroprevalence of *B*. *henselae* infections in cats varies considerably, from none in cold climates (0% in Norway) to high in warm and humid climates (68% in the Philippines) [16]. However, no difference in the prevalence of infection was found between the cold and tropical regions in China. Pet trade may be responsible for the spread of infection in cold regions.

To date, molecular information on Chinese isolates of *B*. *henselae* is still unavailable. Therefore, a MLST analysis was performed on the cat isolates collected in the present study. Hitherto, 26 STs were identified; of these, ST16–26 was defined in a recent study [17]. However, the sequence information of these STs has not been deposited in any public database, which enabled us to assign new STs in the order of their detection. Thus, the new allele combinations in the present study were designed from ST15 in the order of detection. In the present study, 65.4% Chinese isolates belonging to ST1 are associated with human infection. Interestingly, all isolates collected from Midwestern China (Sichuan and Guizhou Provinces) have new allele combinations that resulted in ST16–18. The eBURST analysis confirmed that ST16–18 were phylogenetically related to ST1. Although these new STs belonged to a single clonal complex, the inclusion of the new STs changed the characteristics of the original clonal complex 1, resulting in the designation of ST1 as the primary founder; the former primary founder ST5 was consequently designated as a subgroup founder.

In conclusion, the current study confirms the high prevalence of *B*. *henselae* infection in the cat populations in China. Molecular information from cat isolates in China was first reported, which shows a genetic diversity in the different regions. These results will facilitate an understanding of the population structure and the relationship between humans and cat strains in China in subsequent studies.
